# Acceptance of COVID-19 and Influenza Vaccine Co-Administration: Insights from a Representative Italian Survey

**DOI:** 10.3390/jpm12020139

**Published:** 2022-01-20

**Authors:** Alexander Domnich, Riccardo Grassi, Elettra Fallani, Roberto Ciccone, Bianca Bruzzone, Donatella Panatto, Allegra Ferrari, Marco Salvatore, Maura Cambiaggi, Alessandro Vasco, Andrea Orsi, Giancarlo Icardi

**Affiliations:** 1 Hygiene Unit, San Martino Policlinico Hospital-IRCCS for Oncology and Neurosciences, 16132 Genoa, Italy; bianca.bruzzone@hsanmartino.it (B.B.); andrea.orsi@unige.it (A.O.); icardi@unige.it (G.I.); 2 SWG S.p.A., 34133 Trieste, Italy; riccardo.grassi@swg.it (R.G.); roberto.ciccone@swg.it (R.C.); 3 Seqirus S.R.L., 53035 Monteriggioni, Italy; elettra.fallani@seqirus.com (E.F.); marco.salvatore@seqirus.it (M.S.); maura.cambiaggi@seqirus.com (M.C.); alessandro.vasco@seqirus.com (A.V.); 4 Department of Life Sciences, University of Siena, 53100 Siena, Italy; 5 Department of Health Sciences (DISSAL), University of Genoa, 16132 Genoa, Italy; panatto@unige.it (D.P.); s4270904@studenti.unige.it (A.F.)

**Keywords:** COVID-19, influenza, vaccination, vaccine co-administration, attitudes, survey, Italy

## Abstract

Co-administration of coronavirus disease 2019 (COVID-19) and seasonal influenza vaccines has several advantages, has been advocated by various public health authorities and should be seen as an opportunity to increase the uptake of both vaccines. The objective of this survey was to quantify the acceptance of concomitant COVID-19/influenza vaccination and to identify its correlates in a representative sample of Italian adults. Of 2463 participants, a total of 22.9% were favorable to vaccine co-administration, while 16.6% declared their firm unwillingness to receive both vaccines simultaneously. The remaining 60.5% of subjects could be dubbed hesitant to some degree. Compliance with the primary COVID-19 vaccination schedule (adjusted proportional odds ratio (aOR) = 7.78), previous influenza vaccination (aOR = 1.89) and trust in public health institutions (aOR = 1.22) were the main determinants of positive attitudes toward vaccine co-administration. Other significant correlates included age, sex, perceived disease severity and vaccination risk–benefit, being offered a more personalized influenza vaccine and recent seeking for influenza-related information. In Italy, hesitancy toward COVID-19/influenza vaccine co-administration is common and appears to be higher than hesitancy toward either vaccine administered alone. This pattern is multifaceted and requires specific and tailored strategies, with public health institutions playing the central role.

## 1. Introduction

The ongoing 2021/2022 Northern Hemisphere season is characterized by the co-circulation of severe acute respiratory syndrome coronavirus 2 (SARS-CoV-2) and influenza viruses, although as of December 2021 detections of the latter remain relatively low [1]. At the population level, the “disappearance” of influenza viruses in the previous season determined a progressive waning of acquired immunity; the susceptible fraction of the population is therefore likely to have increased considerably. Moreover, in the current 2021/2022 season, many young children have never been exposed to influenza viruses [2]. Several similarities in the clinical presentation of influenza and coronavirus disease 2019 (COVID-19), the risk of overburdening healthcare systems and the fact that seasonal influenza vaccination (SIV) is among the most effective preventive tools available have prompted a call to increase SIV coverage rates [3,4,5].

It has recently been suggested [6,7,8] that SIV may exert non-specific effects on SARS-CoV-2-related clinical endpoints. In particular, a systematic review and meta-analysis by Wang et al. [7] showed a 14% confidence interval (95% CI: 9–19%) reduction in the odds of testing positive for SARS-CoV-2 in subjects vaccinated against influenza. These non-specific effects may be ascribable to both innate (induction of trained immunity) and adaptive (e.g., cross-immunity and bystander activation) immune-related mechanisms [8]. Indeed, Debisarun et al. [9] reported that, compared with non-vaccinated adults, those who received SIV displayed improved responsiveness of immune cells to heterologous stimuli: SIV modified the anti-SARS-CoV-2 response by reducing interleukin (IL)-1*β* and IL-6 production and increasing IL-1 receptor antagonist (IL-1Ra) release.

The co-administration of COVID-19 vaccine and 2021/2022 SIV may yield some potential benefits, including logistical advantages, cost reduction and the possibility to increase the uptake of both vaccines [10,11]. Preliminary data suggest that the concomitant administration of COVID-19 and SIV vaccines is a feasible option. Specifically, a recent phase IV randomized placebo-controlled trial [12] established that both ChAdOx1 and BNT162b2 COVID-19 vaccines could be safely co-administered with either MF59-adjuvanted or cell-culture-derived SIVs, with no clinically significant increase in adverse events or immunologic inference. Although the available data are limited, the interim guidelines issued by the World Health Organization (WHO) [13] suggest that such co-administration is acceptable. The United States Centers for Disease Control and Prevention (CDC) advocates that COVID-19 vaccines be simultaneously administered with other vaccines, including SIV [14]. Analogously, in October 2021 (i.e., just before the start of the 2021/2022 SIV campaign) the Italian Ministry of Health gave the green light for the vaccine co-administration [15].

Most available research on people’s knowledge, attitudes and practices (KAP) concerning COVID-19 vaccines and/or SIV has focused on socio-structural, contextual and attitudinal determinants of vaccination acceptance, hesitancy or reluctance. As shown by recent systematic reviews [16,17], previous SIV has usually proved to be a strong facilitator of COVID-19 vaccine uptake. On the other hand, very little is as yet known about people’s KAP regarding vaccine co-administration. The objectives of this study were to assess public opinion concerning the simultaneous administration of COVID-19 and SIV vaccines, to quantify the proportion of citizens who are hesitant/reluctant to undergo co-administration and to identify correlates of the willingness to receive both vaccines at the same time. These latter are essential to planning and establishing effective and targeted health promotion interventions to increase immunization coverage rates.

## 2. Materials and Methods

### 2.1. Study Design and Setting

This cross-sectional survey is part of a longitudinal panel study that aims to monitor public opinion toward SIV during the ongoing COVID-19 pandemic. Briefly, data collection is performed 2–3 times a year via computer-assisted web interview (CAWI). During each survey round, we aim to obtain at least 2000 valid responses; this sample size was judged sufficiently powered during the first survey round [18]. The panel is drawn from a well-characterized database of about 60,000 adults (≥18 years) and is representative of the adult Italian population [19,20]. Subjects were sampled by applying a two-stage probabilistic quota method. Specifically, the whole dataset was first grouped into mutually exclusive strata according to sex, age (18–24, 25–34, 35–44, 45–54, 55–64, 65–74 and ≥75 years), geographical macro-area (northeast, northwest, center, south and islands) and size of their municipality of residence. Subsequently, subjects in each stratum were selected randomly. The survey instrument is regularly updated in order to keep pace with the changing epidemiology and public health measures against both COVID-19 and influenza.

The present survey was conducted between 27 October and 12 November 2021. This period was characterized by the following features: (i) the ongoing administration of booster COVID-19 vaccine doses to healthcare professionals, subjects aged ≥60 years and at-risk individuals aged 18–59 years; (ii) the start of the 2021/2022 SIV campaign. Of note, just before the survey, the Italian Ministry of Health authorized the co-administration of COVID-19 and SIV vaccines [15].

Participation in the survey was voluntary, and anonymity was guaranteed. As the replies to all survey items were mandatory, no missing data were expected. The quality of the responses registered was formally checked by analyzing the pattern of responses to signal questions. Specifically, responses to the negatively worded item “Vaccines are a fraud designed to profit the pharmaceutical companies” were compared with those to a positively worded and semantically similar item “Vaccines are crucial to guaranteeing public health and should be mandatory”. Discordant responses to these two items may indicate untruthful or careless responses. These responses were, therefore, excluded from the dataset in a sensitivity analysis.

### 2.2. Study Outcome

The study outcome was the attitude toward simultaneous COVID-19 and SIV vaccine administration and was measured on a four-point Likert scale (“Strongly agree”, “More agree than disagree”, “More disagree than agree” and “Disagree”).

### 2.3. Study Variables

Both individual/socio-structural (sex, age, geographic area of residence, education, income and employment) and attitudinal (different dimensions of KAP concerning immunization, COVID-19 vaccines and/or SIV) independent variables were considered. In particular, the following socio-economic variables were collected: sex, age, highest educational attainment (categorized into six levels according to the International Standard Classification of Education (ISCED) levels adopted in Italy [21], where level 1 corresponds to primary education and level 6 corresponds to advanced research qualifications), employment pattern (employed, student, housekeeper, retired, unemployed, other/prefer not to reply), perceived income (low, lower than average, average, higher than average, high and no personal income) and self-rated health (excellent, very good, good, fair and poor). Participants were also asked whether they had recently searched for information on SIV.

COVID-19 and SIV vaccination status were assessed by means of two items. With regard to SIV, participants were able to select among the following response options: (i) “I have never received SIV”; (ii) “I received SIV in the past, but not in the last 2020/2021 season”; (iii) “I received SIV in the 2020/2021 season for the first time” and (iv) “I received SIV in 2020/2021 and sometimes in the past”. Regarding COVID-19, participants were categorized as follows: (i) fully vaccinated (i.e., those immunized with either two doses of mRNA or ChAdOx1 vaccines or a single dose of Ad26.COV2-S); (ii) partially vaccinated (i.e., those immunized with the first dose of mRNA or ChAdOx1 vaccines); (iii) not vaccinated but having planned/arranged to receive any available COVID-19 vaccine as soon as possible and (iv) individuals who affirmed that they would not receive any available COVID-19 vaccine.

KAP on influenza and/or vaccination were measured on 19 anchored Likert-based items; these are reported in Supplementary Table S1. The comprehensibility of these items was judged sufficient, and a post hoc psychometric evaluation showed acceptable reliability (standardized Cronbach’s *α* of 0.83).

### 2.4. Data Analysis

For descriptive analysis, categorical variables were expressed as proportions with 95% confidence intervals (CIs), while continuous variables were expressed as means with standard deviations (SDs) or medians with interquartile ranges (IQRs). Independent proportions were compared by applying Fisher’s exact test, and the corresponding effect size was expressed as an odds ratio (OR). Ordinal multivariable logistic regression was computed in order to obtain adjusted proportional ORs (aORs) on the association between the willingness to receive COVID-19 and SIV vaccines simultaneously and the independent variables considered. On preliminary analysis, identification of the classes “More agree than disagree” and “More disagree than agree” was suboptimal; these two response options were, therefore, combined into a single category “Unsure”. In summary, the intention to undergo co-administration of COVID-19 and SIV vaccines had three ordered levels (“Strongly disagree”, “Unsure” and “Strongly agree”). On the principle of parsimony, and owing to possible multicollinearity issues, the final model was selected by minimizing the Akaike information criterion (AIC). The goodness of fit, explained variance and discrimination of the model were computed by applying Lipsitz’s test, Nagelkerke’s pseudo-*R*^2^ and *C* index, respectively. Multicollinearity of the final model was checked by quantifying variance inflation factors (VIFs). The independent variable “Age” was treated as continuous, since different categorization rules or the introduction of non-linear terms did not improve the model fit.

Data analysis was performed in R stat packages, version 4.0.3 (R Foundation for Statistical Computing, Vienna, Austria) [22].

## 3. Results

### 3.1. Characteristics of the Study Participants

Of 3630 invitations sent out, a total of 2463 responses (response rate of 67.9%) were received and analyzed. The principal socio-economic characteristics of respondents are reported in Table 1. Briefly, the sample was judged to be geographically representative, men and women were approximately equally distributed, and their mean age was 50.9 (SD 16.8) years, with a range of 18–91 years. Most participants had completed at least secondary education (ISCED level ≥ 4), were employed and claimed to be in good health (Table 1).

### 3.2. Knowledge, Attitudes and Practices concerning Influenza and Vaccination

**Figure 1:**
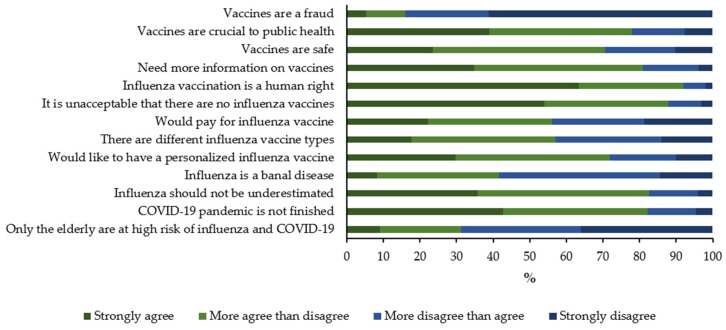
Knowledge, attitudes, and practices on influenza and/or vaccination. Complete wording of the items is reported in Supplementary Table S1. Coronavirus disease 2019, COVID-19.

As shown in Figure 1, most participants valued (influenza) vaccination positively, would like to have more information on vaccines and prefer to have to a more personalized SIV. On the other hand, 41.5% of subjects believed that influenza was a banal disease, and only 56.1% would pay for SIV. Analogously, only 57.0% of respondents agreed to some extent that there were different types of SIV (Figure 1).

**Figure 2:**
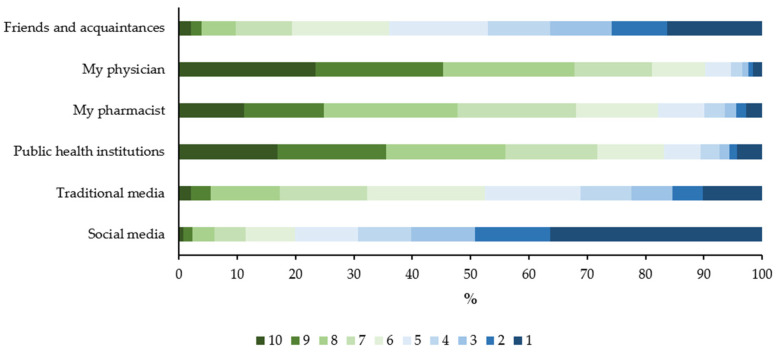
Participants’ trust in different information sources on influenza vaccination (10 indicates the highest trust).

With regard to sources of information on SIV, on a scale of 1-to-10 the most rated source was one’s own physician (median 8 (IQR: 7–8)), followed by public health institutions (median 8 (IQR: 6–8)) and one’s own pharmacist (median 7 (IQR: 6–7)). Social media were deemed the least reputable information source (median 3 (IQR: 1–3)). (Figure 2).

Approximately a quarter (27.7% (95% CI: 26.0–29.5%)) of participants had recently searched for SIV-related information.

### 3.3. Influenza and COVID-19 Vaccination Uptake

As expected, most (85.1% (95% CI: 83.6–86.4%)) participants had completed the primary schedule of COVID-19 vaccination, while 7.8% (95% CI: 6.8–8.9%) declared that they had no intention of being vaccinated. A total of 42.6% (95% CI: 40.6–44.6%) of subjects claimed that they had received the 2020/2021 SIV, while 44.2% (95% CI: 42.2–46.2%) had never been vaccinated against influenza. There was a clear relationship between COVID-19 vaccine and SIV uptake. For instance, the self-declared 2020/2021 SIV uptake was 45.2% (95% CI: 43.1–47.4%) among subjects fully immunized against COVID-19, but only 9.9% (95% CI: 6.1–15.0%) among those who had no intention of being immunized against COVID-19, with an OR of 7.51 (95% CI: 4.62–12.87; *p* < 0.001) (Table 2).

### 3.4. Attitude toward Influenza and COVID-19 Vaccine Co-Administration and Its Correlates

When participants were asked about the co-administration of COVID-19 and influenza vaccines, 22.9% (95% CI: 21.3–24.6%) and 36.1% (95% CI: 34.2–38.0%) of subjects strongly agreed or more agreed than disagreed, respectively. The remaining 16.6% (95% CI: 15.1–18.1%) and 24.4% (95% CI: 22.8–26.2%) replied “Strongly disagree” and “More disagree than agree”, respectively.

From the point of view of the observed effect size, the main determinant (aOR = 7.78) of a positive attitude toward COVID-19/SIV co-administration was completion of the primary COVID-19 vaccination schedule (Table 3). Both partial completion and intention to receive a COVID-19 vaccine as soon as possible were also positive predictors. Analogously, receipt of the previous 2020/2021 SIV and recent information seeking on SIV were associated with significantly higher odds of the willingness to undergo co-administration of COVID-19 and SIV vaccines. Among the structural determinants analyzed, only age and sex were significant predictors: women and older individuals showed lower odds of having a positive attitude toward simultaneous vaccine administration. Beliefs that vaccines are safe and crucial to guaranteeing public health, that influenza is not a banal disease and that the COVID-19 pandemic is not finished and that viral variants continue to circulate were associated with 37–111% higher odds of having a positive attitude toward vaccine co-administration. Interestingly, respondents who would pay for SIV out of pocket (aOR = 1.79) and would prefer to have a more personalized SIV (aOR = 1.55) showed significantly greater odds of the willingness to receive SIV and COVID-19 vaccines simultaneously. By contrast, subjects with a greater propensity to undergo vaccine co-administration were less likely (aOR = 0.60) to need more information on vaccines. Among the different sources of information on SIV, only trust in public health institutions showed a statistically significant association with the study outcome (Table 3). According to Lipsitz’s test, the model fitted the data well (*p* = 0.10), explained 41.8% of variance and displayed acceptable discrimination (*C* = 0.80). No multicollinearity issues emerged (VIFs < 3) nor were significant interaction terms established. 

Finally, following a quality check, a total of 38 responses were judged to be at high risk of untruthfulness or carelessness and were excluded from the dataset in the sensitivity analysis. The results (Supplementary Table S2) showed no substantial changes, although the model fit slightly improved with a reduction of 68 in AIC.

## 4. Discussion

To our knowledge, this is among the first representative surveys aimed at quantifying people’s willingness to undergo concomitant COVID-19/influenza vaccination and identifying its correlates. Concerning the latter, we found that several determinants of vaccine co-administration were shared with those of either COVID-19 or SIV administered separately, although some specific associations also emerged. Together with subsequent official reports on 2021/2022 SIV coverage (expected to be released in summer/fall 2022 and hopefully accompanied by data on the co-administration of the booster COVID-19 dose and SIV), our findings may be useful for planning vaccination campaigns in the next seasons. The future of both the COVID-19 pandemic and its associated immunization policies is largely unknown, and the possibility that SARS-CoV-2 may settle into a seasonal pattern cannot be ruled out; indeed, it seems increasingly likely [23].

The main finding of this study is that the public’s acceptance of COVID-19/SIV co-administration is relatively low. Indeed, only 23% of adult Italians would willingly accept concomitant immunization, while 17% would absolutely not; the remaining majority (approximately 60%) may be dubbed as hesitant to some degree. The number of co-administration-hesitant individuals proved to be higher than that of the SIV-hesitant subjects recorded in our previous studies [11,18] on the acceptance of SIV alone. A similar tendency has been reported with regard to pediatric vaccinations: in Italy, vaccine-hesitant individuals are less favorable toward using combined vaccines and vaccine co-administration [24]. From the point of view of public health, co-administration strategies have several advantages; these, however, are less obvious to laypeople, and communication on the matter may lead to some challenges regarding acceptance. The main public concerns may be the belief that too many vaccines overload the immune system, may be less effective than the same vaccines administered at different time points and may cause a higher number of adverse reactions [25]. It should also be borne in mind that concomitant COVID-19/SIV immunization is a novel recommendation/practice and that even the medical community is not very familiar with this approach. The availability and dissemination of further experimental, observational and pharmacovigilance data; guidelines issued by scientific associations and the personal experience of vaccinating healthcare professionals will probably contribute to increasing public acceptance and the rate of vaccine co-administration. In any case, effective communication tools and approaches will play a crucial role in improving the acceptance of vaccine co-administration.

A recent single-center study by Stefanizzi et al. [26] reported a very high rate of co-administration of the third COVID-19 dose and SIV among healthcare workers. In particular, a total of 60.0% were co-administered both vaccines, while 26.2% and 13.8% of subjects chose to get only the COVID-19 vaccine and SIV, respectively [26]. This comparably high co-administration rate was likely driven by the selected study population of healthcare professionals who received appropriate and effective counseling.

Concomitant vaccine administration is an opportunity to increase immunization coverage, provided that clear clinical guidelines and well-coordinated implementation programs are available. Otherwise, this goal will not be achieved. For instance, during the 2021 Southern Hemisphere influenza season, the COVID-19 immunization program had a negative impact on SIV uptake: in Australia, a marked drop (compared with the 2019 and 2020 seasons) in coverage was reported in all age groups. The exclusion of co-administration was the likely reason for the decrease observed [27]. Unlike the Australian experience, during the ongoing 2021/2022 Northern Hemisphere SIV campaign, clinical guidelines on COVID-19/SIV co-administration have been issued by several public health authorities, including the WHO [13] and CDC [14]. It has been suggested [27] that one of the key actions to maximize the uptake of both COVID-19 and SIV vaccines is for public health authorities to issue clear, consistent, timely and repeated communications on the importance and urgency of having both vaccinations. Our results corroborate this suggestion: among the various information sources on SIV, only people’s trust in public health institutions was associated with a greater likelihood of COVID-19/SIV co-administration. Trust in government and health authorities is a well-known facilitator of vaccine uptake [28], including SIV [29] and COVID-19 [30] vaccines. The degree of trust/mistrust varies according to structural and contextual factors; most people may implicitly trust institutions but at the same time question their competency [31]. It is, therefore, likely that an increase in people’s confidence in public health authorities will reduce vaccination hesitancy at the population level. To achieve this, institutional communication efforts on immunization should be based on best practices of risk communication science and social marketing, which rallies citizens around shared values [32].

We established that both COVID-19 and SIV were independent correlates of a positive attitude toward vaccine co-administration, and the main effect of each one did not depend on the level of the other (no significant interaction was found). The available systematic evidence suggests that previous SIV receipt is a strong predictor of subsequent COVID-19 vaccination acceptance [16,17,33]. Similarly, SIV uptake in previous seasons is associated with subsequent SIV receipt [34,35,36]. Moreover, COVID-19 vaccination status is also a strong correlate of 2021/2022 SIV receipt [11]. In California, a decreasing trend in intentions to receive both COVID-19 and SIV vaccines was observed once the first COVID-19 vaccines were available [37]. Finally, one Italian study [38] found that being hospitalized for COVID-19 during the first pandemic wave was associated with the willingness to receive the SIV but not the COVID-19 vaccine. Causal pathways of this bidirectional COVID-19–SIV relationship, therefore, appear to be very complex and deserve further investigation in a longitudinal modality.

Acceptance of vaccine co-administration was higher among participants who had actively searched for information on SIV in the preceding weeks. In this regard, it has been demonstrated that only active seeking for information is associated with greater SIV uptake, while simple passive exposure to such information is not [39]. We can speculate that, in our sample, active seekers of SIV-related information had a higher probability of finding evidence-based information on COVID-19/SIV co-administration. Indeed, the first search result displayed by Google on typing “Influenza vaccine 2021” shows that “During the 2021/2022 season, the flu shot may be done together with the COVID-19 shot”; this message is located at the website of the Italian Ministry of Health [15]. Again, the role of public health institutions in keeping their websites updated and delivering not only unambiguous but also user-friendly information remains crucial.

Being offered a more personalized SIV was associated with a 55% increase in the odds of having a positive attitude toward COVID-19/SIV vaccine co-administration. The market of available SIVs is highly differentiated [40,41] and some SIV types may be more appropriate for a given population group [42,43]. Indeed, the immune response to SIV depends both on the vaccine type and on a variety of patient characteristics [44]. Similarly, the emerging evidence suggests that the available COVID-19 vaccine types (e.g., mRNA vs. inactivated) present distinct immunogenicity profiles [45], and this may form the basis of more personalized future vaccination schedules. Core theories in economics, psychology and social marketing underline the fact that decision-makers benefit from having more choice [46] and that having more choice is associated with more positive patient outcomes than having no choice [47]. By recognizing that hesitant patients are amenable to modified interventions, personalization of the standardized immunization schedule and service may reduce non-compliance [48].

With regard to COVID-19/SIV vaccine co-administration, an increasing number of randomized controlled trials report data on non-clinically significant inferences (from the point of view of both safety and immunogenicity) of co-administering the available COVID-19 vaccines with age-appropriate SIVs. As of December 2021, these data are available for the cell-based, MF59-adjuvanted (when co-administered with ChAdOx1 [12], BNT162b2 [12] or NVX-CoV2373 [49]), recombinant (when co-administered with either ChAdOx1 or BNT162b2 [12]) and high-dose (when co-administered with mRNA-1273 [50]) SIVs. It is desirable that these co-administration data appear both in summaries of product characteristics (intended primarily for health professionals) and in package leaflets (intended primarily for patients) of both COVID-19 and influenza vaccines.

Other factors able to influence people’s intention to receive COVID-19 and SIV vaccines concomitantly include perception of the risk of disease and its severity, the perceived risk–benefit ratio of vaccination, and some socio-structural determinants, such as age and sex. These factors have been extensively studied as modifiers of either COVID-19 [16,17,30,33] or SIV [34,35,36] vaccine acceptance/refusal. Thus, it is universally accepted that perceived disease susceptibility and vaccination benefits facilitate uptake. Much less clear is the effect of socio-cultural determinants on COVID-19 and SIV vaccination acceptance. Increasing age is usually associated with a higher vaccine acceptance [16,17,30,33,34,35,36]. In our previous survey rounds [11,18], both SIV uptake and acceptance were higher in older individuals. By contrast, in this study an inverse age–acceptance relationship was observed: each 1-year increase in age was associated with a 1.1% decrease in the odds of accepting simultaneous vaccine administration. In the above-mentioned study by Stefanizzi et al. [26], the effect of age on concomitant COVID-19/SIV vaccine administration among healthcare workers was not statistically significant. In England and Wales, SIV and pneumococcal vaccine co-administration was highest in working-age adults, followed by the elderly and children [50]. In our opinion, the lower COVID-19/SIV co-administration acceptance in older subjects may be driven by general tendency of the latter to be more cautious about adopting novel health-related technologies and practices. Similarly, the effect of sex on vaccine acceptance is controversial although it appears that females tend to have lower acceptance rates for both vaccines [16,17,36]. It is even more unclear whether this different acceptance (if any) may generate gender disparities in vaccination coverage. In line with our findings (reversed aOR males vs. females of 1.78 (95% CI: 1.50–2.12)), COVID-19/SIV vaccine co-administration was more prevalent among male healthcare workers with an OR 1.43 (95% CI: 1.22–1.67) [26]. SIV and pneumococcal vaccine co-administration in English/Welsh populations was more frequent (by approximately 6%) in men than in women, and this difference was present in all age-groups, while the between-sex difference in the general pneumococcal vaccination coverage rate was not statistically significant [51]. In sum, the effect of age and gender on vaccine co-administration acceptance should be further investigated.

This study may have several limitations. First, as in all web-based studies, we systematically excluded people with no Internet access. Although Internet penetration in Italy is relatively high, a considerable proportion of citizens still do not use the web. For instance, only 47.3% and 14.9% of the elderly (who are the main target of both COVID-19 and influenza vaccines) aged 65–74 and ≥75 years, respectively, are Internet users [52]. Different patterns of exposure to vaccine-related information may generate some variability in KAP with regard to both COVID-19 and influenza vaccination between Internet users and non-users (e.g., Internet users are likely to be more exposed to anti-vaccination web content). Second, although the study participants were assured about the anonymity of their responses, the social desirability bias cannot be completely ruled out. For instance, in the COVID-19 era, SIV may be seen as more socially favorable, and this may explain the higher than officially reported [53] 2020/2021 SIV uptake. Third, it is unclear whether our results can be generalized to other contexts. Indeed, as our search of the principal scientific databases did not produce any similar study, no direct comparisons could be made. Finally, the cross-sectional nature of the survey did not allow us to establish any causal relationship.

To conclude, as COVID-19/SIV co-administration is a novel public health practice, people’s hesitancy toward this practice is prevalent and seems to be higher than hesitancy toward either vaccine administered alone. Acceptance of vaccine co-administration is driven by a variety of socio-structural, individual and contextual determinants, which should be incorporated into a tailored communication mix, in which public health institutions will play the central role. As shown by the recent Australian experience [27], correct, unambiguous, timely and multi-channel communication and training of healthcare providers in COVID-19 and SIV co-administration is essential in order to maximize the opportunity to vaccinate.

## Figures and Tables

**Table 1:** Socio-economic characteristics of the study participants (*n* = 2463).

Variable	Level	% (*n*)
Sex	Male	47.8 (1177)
	Female	52.2 (1286)
Age, years	18–24	7.9 (194)
	25–34	12.5 (309)
	35–44	15.4 (380)
	45–54	19.8 (488)
	55–64	16.7 (412)
	65–74	21.0 (516)
	≥75	6.7 (164)
Geographic macro-area	Northwest	27.0 (664)
	Northeast	18.9 (465)
	Center	20.1 (496)
	South	22.9 (563)
	Islands	11.2 (275)
Educational level	1	1.5 (38)
	2	8.0 (197)
	3–4	48.2 (1188)
	5	40.5 (997)
	6	1.7 (43)
Employment pattern	Employed	55.4 (1364)
	Student	6.7 (165)
	Housekeeper	8.3 (204)
	Unemployed	5.2 (129)
	Retired	23.5 (580)
	Other/prefer not to reply	0.9 (21)
Perceived income	Low	1.8 (44)
	Lower than average	41.2 (1014)
	Average	30.8 (759)
	Higher than average	7.6 (187)
	High	2.4 (59)
	No personal income	16.2 (400)
Self-rated health	Excellent	11.5 (284)
	Very good	48.3 (1190)
	Good	36.2 (891)
	Fair	3.5 (86)
	Poor	0.5 (12)

**Table 2:** Cross-tabulation of the declared uptake of COVID-19 and seasonal influenza vaccines (*n* = 2463).

Influenza Vaccination	COVID-19 Vaccination, % (*n*)				
	Complete	Partial	Planned	No Intention	Total
Never	35.9 (885)	1.0 (25)	1.5 (36)	5.8 (142)	44.2 (1088)
In the past but not in 2020/2021	10.7 (263)	0.6 (14)	0.7 (18)	1.3 (31)	13.2 (326)
In 2020/2021 but not in the past	10.6 (262)	0.4 (11)	0.7 (17)	0.2 (5)	12.0 (295)
Both in 2020/2021 and in the past	27.8 (685)	1.3 (33)	0.9 (22)	0.6 (14)	30.6 (754)
Total	85.1 (2095)	3.4 (83)	3.8 (93)	7.8 (192)	100 (2463)

Coronavirus disease 2019, COVID-19.

**Table 3:** Multivariable ordinal logistic regression model to predict positive attitude toward COVID-19 and seasonal influenza vaccine co-administration (*n* = 2463).

Variable	Level	aOR (95% CI)	*p*
Sex	Male	Ref	–
	Female	0.56 (0.47–0.67)	<0.001
Age	1-year increase	0.99 (0.98–0.99)	<0.001
Previous influenza vaccination	Never	Ref	–
	In the past but not in 2020/2021	1.09 (0.83–1.44)	0.53
	In 2020/2021 but not in the past	1.52 (1.14–2.04)	0.005
	Both in 2020/2021 and in the past	1.89 (1.49–2.41)	<0.001
COVID-19 vaccination	No intention	Ref	–
	Planned	4.97 (2.70–9.12)	<0.001
	Partial	3.44 (1.81–6.55)	<0.001
	Complete	7.78 (4.91–12.33)	<0.001
Recently searched for influenza vaccination information	No	Ref	–
	Yes	1.38 (1.13–1.69)	0.001
Vaccines are crucial to public health ^1^	Disagree ^2^	Ref	–
	Agree ^3^	1.37 (1.05–1.80)	0.021
Vaccines are safe ^1^	Disagree ^2^	Ref	–
	Agree ^3^	2.11 (1.64–2.70)	<0.001
Need more information on vaccines ^1^	Disagree ^2^	Ref	–
	Agree ^3^	0.60 (0.48–0.75)	<0.001
Would pay for influenza vaccine ^1^	Disagree ^2^	Ref	–
	Agree ^3^	1.79 (1.46–2.19)	<0.001
Would like to have a personalized influenza vaccine ^1^	Disagree ^2^	Ref	–
	Agree ^3^	1.55 (1.25–1.94)	<0.001
Influenza is a banal disease ^1^	Agree ^3^	Ref	–
	Disagree ^2^	1.36 (1.12–1.64)	0.002
COVID-19 pandemic is not finished ^1^	Disagree ^2^	Ref	–
	Agree ^3^	1.32 (1.01–1.73)	0.043
Only the elderly are at high risk of influenza and COVID-19 ^1^	Agree ^3^	Ref	–
	Disagree ^2^	1.20 (0.98–1.47)	0.078
Trust in public health institutions	1-point increase	1.22 (1.16–1.28)	<0.001

^1^ Complete wording of the items is reported in Supplementary Table S1; ^2^ comprises the response options “Strongly disagree” and “More disagree than agree”; ^3^ comprises the response options “Strongly agree” and “More agree than disagree”; aOR, adjusted odds ratio from proportional odds model.

## Data Availability

All relevant data are within the article and Supplementary Materials. Further details may be obtained from the corresponding author upon a reasonable request and prior permission of the study funder.

## Supplementary Materials

The following supporting information can be downloaded at https://www.mdpi.com/article/10.3390/jpm12020139/s1, Table S1: Survey items on knowledge, attitudes and practices (KAP) regarding influenza and/or vaccination, Table S2: Multivariable ordinal logistic regression model to predict positive attitude toward COVID-19 and seasonal influenza vaccine co-administration: Sensitivity analysis.
