# Supplementary material for: Acceptance of COVID-19 and Influenza Vaccine Co-Administration: Insights from a Representative Italian Survey
Source: J Pers Med. 2022 Jan 20;12(2):139. doi: 10.3390/jpm12020139 (PMC8878648; doi:10.3390/jpm12020139)
Supplement: Supplementary file 1 [file jpm-12-00139-s001.zip › Table_S1.pdf]

Table S1. Survey items on knowledge, attitudes, and practices (KAP) regarding influenza and/or vaccination.

**Item**
To what extent do you agree or disagree with the following statement?<br><br>Vaccines are a fraud designed to profit the pharmaceutical companies.<br><br>- Strongly agree;<br>- More agree than disagree;<br>- More disagree than agree;<br>- Strongly disagree.
To what extent do you agree or disagree with the following statement?<br><br>Vaccines are crucial to guaranteeing public health and should be mandatory.<br><br>- Strongly agree;<br>- More agree than disagree;<br>- More disagree than agree;<br>- Strongly disagree.
To what extent do you agree or disagree with the following statement?<br><br>All vaccines are safe.<br><br>- Strongly agree;<br>- More agree than disagree;<br>- More disagree than agree;<br>- Strongly disagree.
To what extent do you agree or disagree with the following statement?<br><br>I need more information on vaccines.<br><br>- Strongly agree;<br>- More agree than disagree;<br>- More disagree than agree;<br>- Strongly disagree.
To what extent do you agree or disagree with the following statement?<br><br>Influenza vaccination is a human right and must be guaranteed for people that would like to have it.<br><br>- Strongly agree;<br>- More agree than disagree;<br>- More disagree than agree;<br>- Strongly disagree.
To what extent do you agree or disagree with the following statement?<br><br>It is unacceptable that there are no influenza vaccines in the future season for people that would like to be vaccinated.<br><br>- Strongly agree;<br>- More agree than disagree;<br>- More disagree than agree;<br>- Strongly disagree.
To what extent do you agree or disagree with the following statement?<br><br>If there were no free-of-charge influenza vaccine, I would pay for it out of my own pocket.<br><br>- Strongly agree;<br>- More agree than disagree;<br>- More disagree than agree;<br>- Strongly disagree.
To what extent do you agree or disagree with the following statement?

On the basis of people's age and health conditions, there are different influenza vaccine types.

- Strongly agree;
- More agree than disagree;
- More disagree than agree;
- Strongly disagree.

To what extent do you agree or disagree with the following statement?

I would be more willing to get a flu shot if it were personalized.

- Strongly agree;
- More agree than disagree;
- More disagree than agree;
- Strongly disagree.

To what extent do you agree or disagree with the following statement?

Influenza is a banal disease: social distancing and wearing masks are sufficient to defeat it.

- Strongly agree;
- More agree than disagree;
- More disagree than agree;
- Strongly disagree.

To what extent do you agree or disagree with the following statement?

Influenza should not be underestimated, influenza virus did not circulate in the last season because more people were vaccinated, while the restrictions adopted further slowed down transmission.

- Strongly agree;
- More agree than disagree;
- More disagree than agree;
- Strongly disagree.

To what extent do you agree or disagree with the following statement?

COVID-19 pandemic is not finished and viral variants continue to circulate; if you get seasonal influenza, you double the risk of having serious complications.

- Strongly agree;
- More agree than disagree;
- More disagree than agree;
- Strongly disagree.

To what extent do you agree or disagree with the following statement?

Only the elderly are at high risk, neither influenza nor COVID-19 is a problem for other age-groups.

- Strongly agree;
- More agree than disagree;
- More disagree than agree;
- Strongly disagree.

Regarding influenza vaccination, on a scale from 1 (not at all) to 10 (completely) how much do you trust information from each of the following sources?

- Friends and acquaintances;
- My physician;
- My pharmacist;
- Public health institutions;
- Traditional media (radio/TV/newspapers);
- Social networks.
