# Supplementary material for: Acceptance of COVID-19 and Influenza Vaccine Co-Administration: Insights from a Representative Italian Survey
Source: J Pers Med. 2022 Jan 20;12(2):139. doi: 10.3390/jpm12020139 (PMC8878648; doi:10.3390/jpm12020139)
Supplement: Supplementary file 1 [file jpm-12-00139-s001.zip › Table_S2.pdf]

Table S2. Multivariable ordinal logistic regression model to predict positive attitude towards COVID-19 and seasonal influenza vaccine co-administration: Sensitivity analysis (n = 2,425).

Variable	Level	aOR (95% CI)	*p*
Sex	Male	Ref	–
	Female	0.55 (0.46–0.66)	<0.001
Age	1-year increase	0.99 (0.98–0.99)	<0.001
Previous influenza vaccination	Never	Ref	–
	In the past, but not in 2020/21	1.04 (0.79–1.37)	0.77
	In 2020/21, but not in the past	1.53 (1.14–2.05)	0.005
	Both in 2020/21 and in the past	1.97 (1.54–2.52)	<0.001
COVID-19 vaccination	No intention	Ref	–
	Planned	5.65 (3.03–10.53)	<0.001
	Partial	3.90 (2.02–7.51)	<0.001
	Complete	8.47 (5.30–13.54)	<0.001
Recently searched for influenza vaccination information	No	Ref	–
	Yes	1.40 (1.15–1.71)	0.001
Vaccines are crucial to public health [1]	Disagree [2]	Ref	–
	Agree [3]	1.38 (1.05–1.81)	0.020
Vaccines are safe [1]	Disagree [2]	Ref	–
	Agree [3]	2.17 (1.68–2.79)	<0.001
Need more information on vaccines [1]	Disagree [2]	Ref	–
	Agree [3]	0.60 (0.48–0.75)	<0.001
Would pay for influenza vaccine [1]	Disagree [2]	Ref	–
	Agree [3]	1.78 (1.45–2.18)	<0.001
Would like to have a personalized influenza vaccine [1]	Disagree [2]	Ref	–
	Agree [3]	1.58 (1.27–1.97)	<0.001
Influenza is a banal disease [1]	Agree [3]	Ref	–
	Disagree [2]	1.35 (1.11–1.64)	0.002
COVID-19 pandemic is not finished [1]	Disagree [2]	Ref	–
	Agree [3]	1.33 (1.01–1.75)	0.039
Only the elderly are at high risk of influenza and COVID-19 [1]	Agree [3]	Ref	–
	Disagree [2]	1.16 (0.94–1.42)	0.17
Trust in public health institutions	1-point increase	1.21 (1.15–1.27)	<0.001

[1] Complete wording of the items is reported in Supplementary Table S1; [2] Comprise response options "Strongly disagree" and "More disagree than agree"; [3] Comprise response options "Strongly agree" and "More agree than disagree"; aOR, adjusted proportional odds ratio
